# In vitro activity of vancomycin, quinupristin/dalfopristin, and linezolid against intact and disrupted biofilms of staphylococci

**DOI:** 10.1186/1476-0711-4-2

**Published:** 2005-01-07

**Authors:** Mohamed El-Azizi, Suma Rao, Termkiat Kanchanapoom, Nancy Khardori

**Affiliations:** 1 Division of Infectious Diseases, Southern Illinois University School of Medicine, Springfield, IL 62794, USA

**Keywords:** Biofilm, disrupted biofilm, vancomycin, quinupristin/dalfopristin, linezolid.

## Abstract

Shed cells or disrupted parts of the biofilm may enter the circulation causing serious and very hard to treat biofilm-associated infections. The activity of antimicrobial agents against the shed cells/disrupted biofilms is largely unknown.

**Methods:**

We studied the *in vitro *susceptibility of intact and disrupted biofilms of thirty clinical isolates of methicillin-resistant and methicillin–susceptible *Staphylococcus aureus *(MRSA and MSSA) and *Staphylococcus epidermidis *to vancomycin, quinupristin/dalfopristin, and linezolid and compared it to that of the suspended (planktonic) cells.

**Results:**

Bacteria in the disrupted biofilms were as resistant as those in the intact biofilms at the minimum inhibitory concentrations of the antibiotics. At higher concentrations, bacteria in the disrupted biofilms were significantly (*P *< 0.001) less resistant than those in the intact biofilms but more resistant than the planktonic cells. Quinupristin/dalfopristin showed the best activity against cells of the disrupted biofilms at concentrations above MICs and vancomycin, at 500 and 1,000 μg/ml, was significantly more active against the biofilms of MRSA and *S. epidermidis*

**Conclusion:**

The difficulty of treating biofilm-associated infections may be attributed not only to the difficulty of eradicating the biofilm focus but also to the lack of susceptibility of cells disrupted from the biofilm to antimicrobial agents.

## Introduction

Gram-positive infections have become a serious problem, especially in the nosocomialsetting, and treatment of these infections is complicated by the emergence of multidrug-resistant pathogens [1]. Infections caused by *Staphylococcus aureus *and *Staphylococcus epidermidis *are among the most frequent causes of both healthcare-associated and community-onset infections [2]. Staphylococci cause a large percentage of infections by forming biofilms on medical implants, damaged tissues, and most commonly on indwelling vascular catheters [3-7].

Biofilm-associated infections are becoming more common, and occur largely because of the increase in the use of indwelling medical devices. Central venous catheters (CVCs), for example, are inserted in more than 20 million hospitalized patients in the United States alone each year [8]. The mortality rate due to CVC-related bloodstream infections is estimated to be 12–25%, with additional healthcare costs in the order of $33,000 to $35,000 per event [9]. The predominant microorganisms associated with CVC-related infections are *Staphylococcus epidermidis *and *Staphylococcus aureus *where they are often found in biofilms upon removal of the devices [10-12]. Biofilm associated infections are difficult to treat due to the inherent antibiotic resistance of the sessile bacteria [4,6,13]. A number of factors contribute to this resistance such as a slow growth rate [14,15], failure of the agent to penetrate the biofilm [16,17], physiological changes and gene expression or repression due to the biofilm mode of growth [18,19]. Other factors such as age of the biofilms [20], production of extracellular polymeric substance (EPS) [21-23], and presence of biomaterials [24] also play a role in decreasing susceptibility of the bacteria within the biofilms to antimicrobial agents.

Routinely, the diagnostic laboratories report the susceptibilities done on planktonic bacteria only. Although many studies have focused on the antimicrobial susceptibility of bacteria grown in biofilms [25-28], none of these studies included bacteria that disrupted from the biofilms. Disruption of the biofilm can occur during the removal of colonized catheters or during fluid infusion through them. The result is the entrance of bacteria or groups of bacteria shed from the biofilm into circulation causing bloodstream infections. These bacteria may not belong to either the planktonic or the biofilm phase and consequently may have a different pattern of antimicrobial susceptibility. For this reason, we studied the *in vitro *susceptibility of intact and disrupted biofilms of MRSA, MSSA and *S. epidermidis *to vancomycin, quinupristin/dalfopristin, and linezolid and compared it to the susceptibility patterns of the same bacteria in suspension.

## 1. Materials and Methods

Unless otherwise indicated, all chemicals (analytical grade) were purchased from Sigma Chemical Co., St. Louis, Missouri, USA.

### Antibiotics

Vancomycin (VAN) was purchased from Sigma Chemical Co., St. Louis, Missouri, USA. Quinupristin/dalfopristin (Q/D) was provided by Rhone-Poulenc Rorer, Collegeville, PA, USA and Linezolid (LNZ) was provided by Pharmacia & Upjohn, Kalamazoo, MI, USA.

### Microorganisms

Ten isolates each of MRSA, MSSA, and *S. epidermidis *were used in this study. The microorganisms are clinical isolates from patients with blood stream infections which were provided by the microbiology laboratories at St. John's Hospital, Springfield, Illinois. These isolates were screened for biofilm formation on polystyrene microliter plates as previously described [27].

### Antimicrobial susceptibility in suspension

The minimum inhibitory concentrations (MICs) of the antibiotics were determined by using the broth microdilution technique as described by the National Committee for Clinical Laboratory Standards (NCCLS) [29]. The minimum bactericidal concentrations (MBCs) were determined by mixing the contents of each well at MIC and higher concentrations. Ten-microliter portions were then taken from each well and streaked onto the surface of blood agar. After 24 h incubation, the number of colony forming units per milliliter (CFU/ml) were counted and the MBCs, defined as the concentration at which 99.9% of bacteria was killed, were determined. The MIC_90 _and MBC_90 _obtained in susceptibility testing on planktonic bacteria were used in interpreting the results of the experiments with intact and disrupted biofilms.

### Biofilm formation and quantification

To form biofilms, 100 μl portions of Tryptic Soy Broth (TSB) (Difco laboratories, Detroit, MI, USA) containing 1 × 10^6 ^CFU/ml of the microorganisms were delivered to flat bottom 96 polystyrene plates (Falcon No. 353072, Becton Dickinson and Company, Franklin Lakes, NJ, USA). After 24 h incubation at 37°C, the supernatants were aspirated and the remaining biofilms were washed twice with distilled water. TSB with or without the antibiotics at MIC values or at 50,500, or 1,000 μg/ml was added to the wells. Biofilms in the plates used for disrupted wells were then dislodged by using sterile wooden sticks and all plates were incubated again for 24 h. Plates with disrupted biofilms were centrifuged at 3,000 rpm for 15 min. to sediment the biofilm particles and all plates were then cautiously aspirated. The intact biofilms and the sediments of the disrupted biofilms were then determined by using a modified colorimetric assay previously described by Roslev & King [30]. On this assay, bacteria with an active electron transport system reduce the tetrazolium salt (redoxdye) to water soluble orange formazan product. Briefly, 100 μl lactate Ringers solution containing tetrazolium sodium 3'-{1- [(phenylamino)-carbonyl]-3,4-tetrazolium}-bis (4-methoxy-6-nitro) benzene sulfonic acid hydrate (XTT) (0.5 gm/L) and menadione (1 μM) was added to the intact biofilms and the disrupted biofilm sediments. The contents of the plates were mixed via plate shaker (Lab-Line Instruments Inc., Melrose Park, IL, USA) for 5 min followed by incubation for 1 hat 37°C in the dark. Plates with disrupted biofilm were first centrifuged for 15 min at 4°C, and the supernatants containing the soluble colored formazon were transferred to new plates. The intensity of the color was then measured via micro plate reader (Multiscan Plus, Thermosan Systems, Finland) at 490 nm and compared to that of drug-free wells. For plates with intact biofilms, the intensity of the color of the soluble formazan was measured directly and compared to drug-free wells.

### Confocal Scanning Laser Microscopy (CSLM)

One-milliliter portions of TSB containing 1 × 10^6 ^CFU/ml of *S. epidermidis *isolate (SE6) were used to inoculate sterile plastic cover slips placed in a 4 well multidish (Nunc No. 176740, Roskilde, Denmark). After 24 h incubation at 37°C, the cover slips were moved to new plates and washed twice with distilled water. Fresh TSB (1 ml) with 500 μg/ml of vancomycin was added to the wells. Biofilms on cover slips designed to study the disrupted biofilms were then carefully dislodged. After incubation for another 24 h, the biofilms (intact and disrupted) were stained with LIVE/DEAD BacLight bacterial viability stain (Molecular Probes, Eugene, OR, USA) following manufacturer's instructions. The biofilms (intact and disrupted) were then examined by Olympus Fluoview CSLM (model IX 70, Olympus America Inc. NY, USA).

In another set of experiments, the susceptibility of planktonic cells was examined by growing the bacteria in 1 ml portions of TSB. After 24 h, the bacterial suspensions were centrifuged at 10,000 rpm for 10 minutes, washed twice with sterile distilled water and finally dispersed in 1 ml portions of fresh TSB with the antibiotic. After another 24 h of incubation, the bacteria were stained and examined as previously mentioned.

### Statistical Analysis

The mean and S.D. were calculated from the results of 10 isolates of each of the Staphylococcal species. One-way analysis of variance (ANOVA) was used to determine the differences between various antibiotic treatments. Tukey's pair comparison test was used at the chosen level of probability (*P *< 0.05) to determine significance difference between means.

## Results

**Figure 1:**
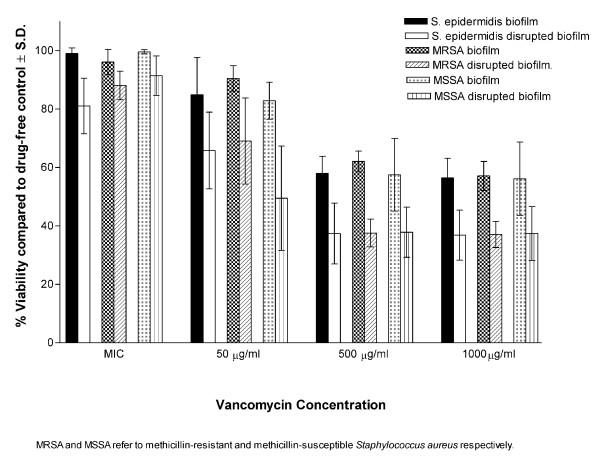
Susceptibility of intact and disrupted biofilms of MSSA, MRSA, and *S. epidermidis *at different concentrations of vancomycin.

**Figure 2:**
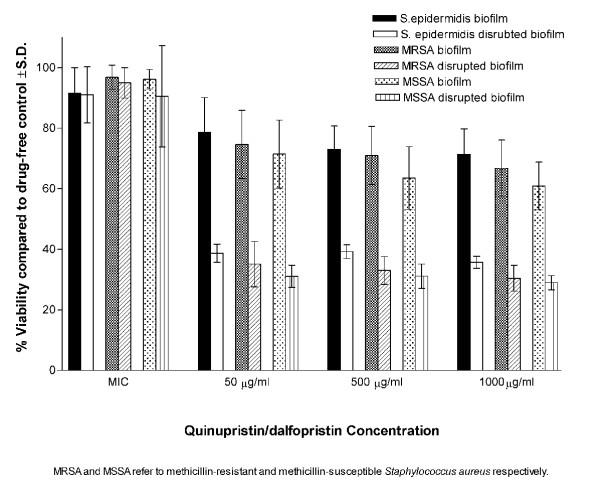
Susceptibility of intact and disrupted biofilms of MSSA, MRSA, and *S. epidermidis *at different concentrations of quinupristin/dalfopristin.

**Figure 3:**
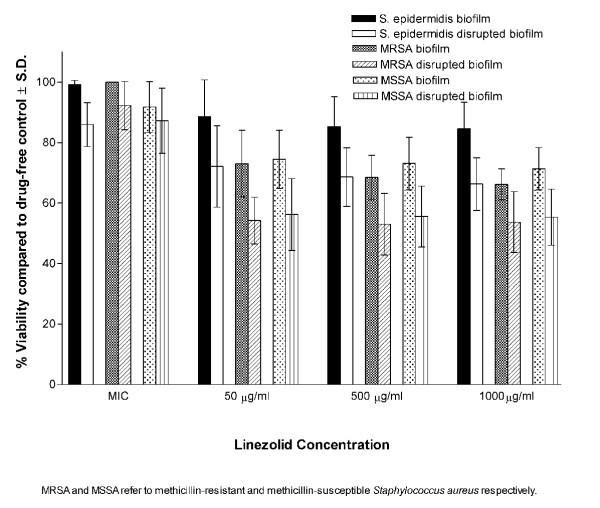
Susceptibility of intact and disrupted biofilms of MSSA, MRSA, and *S. epidermidis *at different concentrations of linezolid.

In suspensions, all isolates were susceptible to all antibiotics tested (Table 1). At MICs, the antibiotics showed very little effect on the viability of bacteria within the biofilms (intact or disrupted). At higher concentrations (50,500 and 1000 μg/ml), the biofilms of all isolates were significantly (P < 0.001) less susceptible to the antibiotics compared to disrupted biofilms (Figures 1, 2, 3). Linezolid was less active than quinupristin/dalfopristin and vancomycin in killing the bacteria, especially in the biofilms.

**Table 1:** Susceptibility of the tested isolates to vancomycin, quinupristin/dalfopristin and linezolid in suspension.

**Microorganism ^a^**	**Antimicrobial agents (μg/ml)**		
			
	**Vancomycin**	**Quinupristin/dalfopristin**	**Linezolid**
Methicillin-susceptible *S. aureus *(MSSA)			
MIC Range	0.50–1	0.125–0.25	1–2
MIC_90_	1	0.25	2
MBC Range	8–16	8	>64
MBC_90_	8	8	>64
Methicillin-resistant *S. aureus *(MRSA)			
MIC Range	0.5–1	0.25–50	1–2
MIC_90_	1	0.50	2
MBC Range	2–16	4–16	>64
MBC_90_	8	16	>64
*S. epidermidis*			
MIC Range	2–4	0.06–2	0.50–1
MIC_90_	2	0.50	1
MBC Range	2–16	0.25–16	>64
MBC_90_	8	8	>64

^a ^The MIC defined as the minimum concentration of antibiotic at which growth was completely inhibited while the MBC defined as the minimum concentration at which 99.9% of bacteria was killed.

**Figure 4:**
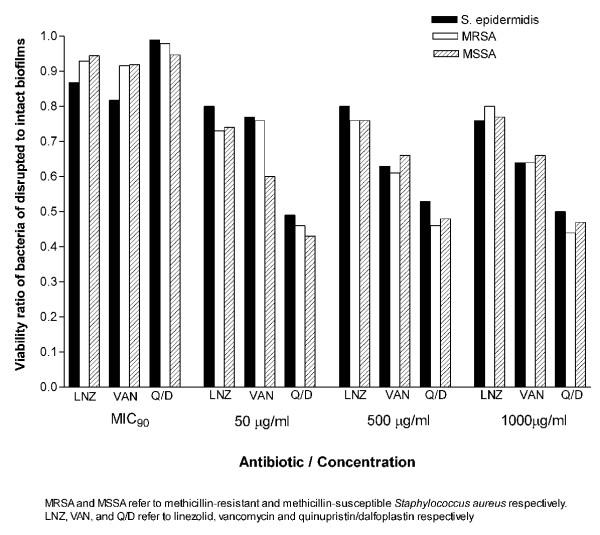
Viability ratios of MSSA, MRSA, and *S. epidermidis *of disrupted to intact biofilms at different concentrations of vancomycin, quinupristin/dalfopristin and linezolid.

Quinupristin/dalfopristin showed the best activity against cells of the disrupted biofilms at concentrations above MICs. It was also more active than vancomycin against biofilms of both *S. aureus *and *S. epidermidis *at 50 μg/ml. Vancomycin at 500 and 1,000 μg/ml, was significantly more active against the biofilms of MRSA and *S. epidermidis *but not MSSA. Killing of the bacterial cells in intact or disrupted biofilms by quinupristin/dalfopristin and linezolid was independent of antibiotic concentrations over the range of 50–1,000 μg/ml, but for vancomycin, this was observed at higher concentration range (500–1,000 μg/ml). The ratios of viability of disrupted biofilms to that of intact biofilms were calculated for the isolates with each antibiotic concentration (Figure 4). The ratio values were similar for the three antibiotics at MICs. At other concentrations, the highest viability ratio was observed with linezolid and the lowest with quinupristin/dalfopristin.

**Figure 5:**
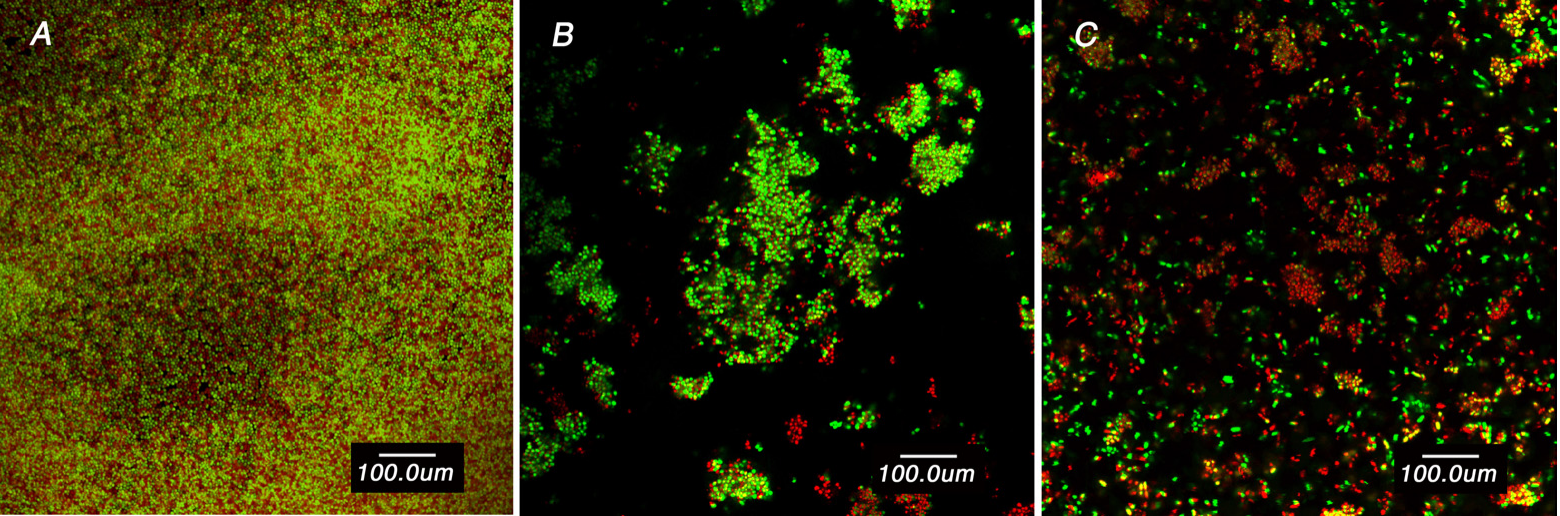
CSLM images of S. *epidermidis *(SE6) intact biofilms (A), disrupted biofilm (B) and planktonic cells (C) on plastic coverslips after incubation for 24 h with 500 μg/ml of vancomycin. The bacterial cells were stained with LIVE/DEAD BacLight bacterial viability stain to directly visualize the effects of the antibiotic. The green fluorescence reflects processing of the dye by metabolically active cells while the red fluorescence is characteristic of dead cells. Note that while the green fluorescence was considerably more prominent in the intact biofilm image, the disrupted biofilm does display more green fluorescence than the planktonic cells. Also, note that the disrupted biofilm consists of large clumps and aggregates compared to the typical clusters of planktonic cells.

CSLM (Figure 5) demonstrated resistance of the intact biofilms to vancomycin, indicated by large number of viable cells, and resistance of the disrupted biofilm compared to the planktonic cells. It is also clear that the disrupted biofilm consists of clumps of larger size compared to that of the planktonic cells.

## Discussion

Coagulase-negative staphylococci and *S. aureus *(mostly methicillin-resistant) are among the leading causes of nosocomial blood stream infections in the USA [31] with a crude mortality of 21–25% respectively [32]. *S. aureus *accounted for up to 13% of isolates recovered from patients with nosocomial infections from 1979 through 1995, and the percentage has increased in recent years [33,34]. It has been estimated that 65% of nosocomial infections are biofilm associated [5,35]. *S. epidermidis *is a common cause of blood stream infections associated with indwelling medical devices. *S. aureus*, in addition to causing blood stream infections, is a significant cause of tissue infections such as pneumonia and osteomyelitis [5].

Three phases of bacteria were used in this study; planktonic, biofilms and disrupted biofilms. Planktonic cells were used for determination of MICs and MBCs. This phase of bacteria has routinely been used as gold standard for determination of susceptibility of bacteria and prediction of clinical efficacy of antimicrobial agents. Planktonic cells grown in *in vitro *batch cultures are usually in nutrient-rich medium. Bacteria grown in biofilms differ greatly from the same organisms grown in suspensions by having different growth characteristics and taking up nutrients and drugs differently [36,37]. When a biofilm is at steady state, cells are shed from it at a constant rate [38]. These cells may enter into circulation and cause blood stream infection. Biofilm associated infections are 10 to 1,000 times more resistant to the effects of antimicrobial agents [5,35,39]. Bacteria are shed through biofilm disruption, which may result in entrance of biofilm pieces into circulation causing systemic infections. The question that needed to be answered is whether these cells behave as planktonic cells or as biofilm in regards to susceptibility to antimicrobial agents.

We used 24 h incubation for interaction with antimicrobial agents in keeping with the procedures followed in diagnostic laboratories. Although it is possible that during this long incubation period the disrupted biofilm could have re-adhered to the polystyrene, we believe this was not the case. This is based on finding in our and other laboratories that antimicrobial agents are able to prevent the adherence of bacteria when exposed to them prior to formation of the biofilm [25-27]

All isolates in suspension were susceptible to the antibiotics as determined by NCCLS guidelines. Vancomycin and quinupristin/dalfopristin were capable of killing 99.9 % of the bacteria in suspension at concentrations up to 16 μg/ml, while linezolid, a bacteriostatic antibiotic, did not show such effect even at the maximum concentration used. At the MICs, the antibiotics exerted little effect on the viability of the intact and disrupted biofilms. This was expected because even in suspension the MIC_90_s were 4–8 times lower then the MBC_90_s for vancomycin and 16–32 times lower for quinupristin/dalfopristin. At higher concentrations, although the intact biofilms were significantly more resistant than the disrupted biofilms, the recalcitrance of the latter was clear. The antibiotic concentrations were 100–4,000 times the MIC_90_s for quinupristin/dalfopristin and 25–1,000 times the MIC_90_s for vancomycin and linezolid. None of these concentrations were able to kill 99.9 % of the microorganisms in the disrupted phase. This was further demonstrated by examining the viability of *S. epidermidis *cells in the three phases in the presence of vancomycin by using CSLM. The susceptibility of the disrupted biofilm lies between the highly resistant biofilm and the susceptible planktonic cells. This may be attributed to the fact that disrupted biofilms consist of fragments that may retain some features of the intact biofilms. It is obvious from the images that the disrupted biofilm consists of large clumps and aggregates compared to the typical clusters of planktonic cells. The semi quantitative assessment of viability by CSLM was similar to those obtained by the colorimetric assay.

Killing of the bacterial cells in intact or disrupted biofilms by quinupristin/dalfopristin and linezolid was independent of antibiotic concentrations over the range of 50–1000 μg/ml and over a higher range of vancomycin concentrations (500–1,000 μg/ml). Hamilton-Miller & Shah [40] found that killing of *S. epidermidis *in the biofilm by quinupristin/dalfopristin was independent of antibiotic concentrations over the range of 20–200 times the MIC. This lack of dose response at high concentrations favors the hypothesis that the bacteria disrupted from the biofilm are more or less similar to those in the biofilm rather than the bacteria in suspension.

For better comparison, the viability ratios of the disrupted to the intact biofilms were calculated at different concentrations of the antibiotics. Linezolid was less efficient in killing bacterial cells in intact or disrupted biofilms which explains its highest viability ratio. Quinupristin/dalfopristin, with the lowest viability ratio, was more active against the cells of the disrupted biofilms at concentrations above MICs for both *S. aureus *and *S. epidermidis*. It was also more active than vancomycin against biofilms of both *S. aureus *and *S. epidermidis *at 50 μg/ml. On the other hand, vancomycin at 500 and 1,000 μg/ml, was significantly more active against the biofilms of MRSA and *S. epidermidis *but not MSSA. It has been reported that vancomycin accumulates at high concentration in the biofilms of gram positive bacteria especially *S. epidermidis *compared to linezolid [41]. This may be attributed to the ability of glycopeptides to bind to exopolysaccharides produced by the bacteria. However, such high concentrations of vancomycin or quinupristin/dalfopristin are not achievable in clinical practice.

In general, our data show that Quinupristin/dalfopristin is more active than vancomycin and linezolid against the disrupted biofilms and there was no difference between methicillin-susceptible and methicillin resistant staphylococci. We conclude that the difficulty in treating the infections related to indwelling medical devices may not be only due to lack of eradication of the cells in the biofilm phase, but also due to resistance of bacteria disrupted from the biofilm.

## Authors' contributions

ME did the biofilm work and participated with NK in the design of the study, review and interpretation of the data and discussion. SR and TK participated in the determination of MICs and MBCs of the antibiotics. All authors read and approved the final manuscript.
